# Brazilian Green Propolis Compared to Miconazole Gel in the Treatment of *Candida*-Associated Denture Stomatitis

**DOI:** 10.1155/2013/947980

**Published:** 2013-05-02

**Authors:** Hermínia Marques Capistrano, Eliene Magda de Assis, Rosana Maria Leal, Maria Eugênia Alvarez-Leite, Sylvie Brener, Esther Margarida Alves Ferreira Bastos

**Affiliations:** ^1^Oral Pathology Laboratory and Stomatology, Dentistry Department, Pontifícia Universidade Católica de Minas Gerais, Dom José Gaspar Avenida, 500, 30535-901 Belo Horizonte, MG, Brazil; ^2^DDS Private Practice in Dentistry, Brazil; ^3^Microbiology Laboratory, Dentistry Departament, Pontifícia Universidade Católica de Minas Gerais, Brazil; ^4^Researcher of FUNED (Fundação Ezequiel Dias) of Minas Gerais, Brazil

## Abstract

*Aim*. To evaluate the efficacy of Brazilian green propolis in comparison to miconazole gel in the treatment of *Candida*-associated denture stomatitis. *Methods*. Forty-five denture stomatitis patients, with palatal mucosa erythema levels classified according to Newtons's criteria and with positive culture to *Candida* spp., were randomly divided into three treatment groups: 15 received miconazole gel 2%, 15 received propolis gel 2,5%, and 15 received propolis 24% for mouthwash. After four daily use lasting two weeks, they were reexamined for the denture stomatitis degree and for a second culture of *Candida*. The Wilcoxon's test was applied to compare the results of clinical classification of the denture stomatitis and the *Candida* spp. colonies numbers, before and after each treatment. The Kruskall-Wallis's test was used to compare efficacy among the three treatment groups. *Results*. There were a significant reduction or complete remission of denture stomatitis (*P* < 0.05) and a significant decrease of *Candida* colonies for the three groups (*P* < 0.05). There was no difference in the efficacy among the treatment groups (*P* > 0.05). *Conclusion*. Brazilian green propolis has a similar effect as miconazole in the treatment of *Candida*-associated denture stomatitis being an alternative in the therapeutics of this condition.

## 1. Introduction

Denture stomatitis is a common inflammatory lesion in the palatal mucosa of denture wearers who presenting a erythema of variable intensity and extension. Prevalence studies varying between 10% and more than 65% [1, 2]. This etiology is multifactorial involving predisposing factors, for example, denture stability, oral and denture hygiene, lasting use, systemic factors like immunologic and endocrinal diseases, nutritional deficiency, and some medications including corticoids, antibiotics, and immunossupressors [3, 4]. An association between denture stomatitis and *Candida *spp., specially *Candida albicans,* has been reported [2, 5–7].

The treatment for denture stomatitis includes meticulous denture hygiene and the reduction of local and general predisposing factors. The association of these procedures with topic antifungal therapy showed good results in the reduction of the palatal mucosa inflammation and decreasing of yeast number of *Candida *spp. in the palate and in denture fitting [2, 8, 9]. The most common synthetic drugs used are imidazole related compounds such as miconazole, polyenic derivatives such as nystatin and others [2, 3, 5, 10]. 

Miconazole is an antifungal for topical oral administration and has been showing a good effect in therapeutic or prophylactic treatment of *Candida*-associated denture stomatitis, reducing erythema [2, 11], and decreasing colonies of *Candida *spp. [2, 5]. 

Propolis is a resinous material collect by bees from various plants, and this chemical composition depends on its origin area [12–14]. Propolis has been used as an anti-inflammatory purpose in folk medicine since early times especially in Europe [13]. Extract of propolis contains a wide variety of components like flavonoids and phenolic acids. The flavonoids in propolis, mainly pinocembrin, have been considered to be responsible for its inhibitory effect on *Candida* [15], but only traces of these compounds have been found in propolis of South American origin [16], indicating that this effect could be due to a different class of compounds such as the high content of aromatic acids and possibly the presence of amyrins [17] that is known to possess several therapeutical activities [18, 19]. 

Several new compounds have already been identified in Brazilian green propolis samples [20, 21], and studies have attesting its biological effects like microbicidal, anti-inflammatory, antioxidative, anticancer, and cytotoxic activities and their therapeutics uses [13, 16, 18, 22, 23]. Antifungal activity is one of the most extensively investigated biological actions of propolis [22]. Thus, the aim of this study was the comparison of the efficacy of a new form of Brazilian green propolis, using a formula propylene glycol contrasting it with miconazole gel in the topical treatment of *Candida*-associated denture stomatitis. 

## 2. Material and Methods

This study was approved by Ethics Committee of the Pontificia Universidade Catolica de Minas Gerais (CEP/PUCMinas, protocol number: CEP 0022.0.213.000-05. Address: Avenida Dom José Gaspar 500, Dom Cabral-Belo Horizonte, MG, Brazil; June 9th, 2005). All participants were informed about the study objectives and signed a declaration of informed consent before the start of the study.

### 2.1. Subject Selection and Samples Collection

At baseline, eighty denture wearers individuals, presenting clinical denture stomatitis, were selected randomly among patients of the Pathology Clinic of a Dentistry School (Departamento de Odontologia da Pontificia Universidade Católica de Minas Gerais (DOPUC) Minas), Brazil. Thirty more subjects with normal palatal mucosa (15 denture users and 15 having natural teeth) were also selected to compare the count of colony-forming units (CFUs) of *Candida *spp. among subjects with and without denture stomatitis. 

Patient inclusion criteria were (1) denture stomatitis covered the palatal mucosa; (2) diagnosed candidiasis confirmed by microbiologic cultures from the erythematous palatal mucosa of the denture wearers. The exclusion criteria were as follows: (1) no patient user of antimycotics, antibiotics, or anti-inflammatory during 2 months before selection; (2) no medication that caused hyposalivation or xerostomia. Swabs were used to collect yeast samples from the denture underlying palatal mucosa of the 110 individuals, with (*n* = 80) and without (*n* = 30) denture stomatitis. Swabs were plated onto Sabouraud dextrose agar (Difco) containing chloramphenicol (100 mg/L) and incubated aerobically at 28°C. Isolation and identification of the species were performed after 48 hours of incubation. Before isolation, the number of CFUs was determined, and morphological characteristics were observed. All isolates were identified with the aid of a germ-tube test (Reynolds-Braude effect) with positive test and presumptive diagnose of *Candida albicans*. 

### 2.2. Study Groups, Data Collection and Clinical Evaluation

Out of 85 patients with clinical presentation of denture stomatitis, only 45 had *Candida* spp. positive microbiological tests, and these patients were included in the study group. All individuals with health palatal mucosa either denture users or with natural teeth (*n* = 30) had no yeast colony growth in culture. 

At start, personal data and medical history of the 45 individuals of the study groups were obtained. Patients were inquired about habits like sleeping with denture, age of dentures, last change of the prosthesis, and how their denture hygiene was done. All of them had instructions about denture hygiene. Clinical examination and smears of palatal mucosa for culture were made by only one examiner, an oral pathologist following the biosafety norms. The palatal mucosa erythema levels were classified according to Newtons's criteria [24], and photographs of their palatal mucosa were taken by the same examiner. 

The patients were randomically allocated in three parallel treatment groups. Group I was treated with topical use miconazole gel 2% (*n* = 15; mean age 62.5 ± 13.5 years; 3 men and 12 women). Group II was treated with topical use propylene glycol Brazilian green propolis gel 2.5% (*n* = 15; mean age 57.7 ± 13.2 years; 2 man and 13 women). Group III received propylene glycol Brazilian green propolis 24%, in form of mouthwash (*n* = 15; mean age 62.0 ± 5.5 years; 2 men and 13 women). The propylene glycol extract of Brazilian green propolis used in two different groups had the objective of testing its most efficient and best-tasting form, gel or mouthwash, once this is a pilot study.

### 2.3. Treatment Posology

The standard protocol used for the three groups included previous hygiene of denture. Following, patients of the Groups I and II applied miconazole gel and propolis gel, respectively, (5 mL or one teaspoon) with a cotton swab in the inner surface of denture, which was immediately placed in the mouth, and surplus gel was spat out. Patients of Group III made mouthwash with the solution of propolis (5 mL) during a minute and then spat out. All the treatments groups have used four daily applications, during 14 days. The patients were asked to maintain their usual regime for wearing their dentures. After a week the patients were reexamined to verify any intolerance to the medications and how their denture hygiene was. The treatment was given by another oral medicine examiner.

After the treatment the same first oral medicine examiner, in a double-blind way, evaluated all the patients of the three study groups for a second classification for denture stomatitis, new photographs, and a new swab of palatal mucosa, following the same protocol used before the treatments.

The glycolic extract of propolis applied in this research was formulated and provided by Fundação Ezequiel Dias, in Belo Horizonte, MG, Brazil. *In vitro* tests were done to establish the better concentration and the therapeutic efficacy of the propylene glycol extract of Brazilian green propolis, according to the National Committee for Clinical Laboratory Standards [25].

### 2.4. Statistical Analysis

To compare the results of clinical classification of the denture stomatitis and the *Candida *spp. colonies number, before and after each treatment, Wilcoxon's test was applied. The Kruskall-Wallis's test was used to compare the efficacy among the three treatment groups (Biostat, 4.0). The considered significance level was *P* < 0.05.

## 3. Results

Data of the interview showed that denture hygiene was done in a similar way by all denture wearers, including the 35 patients with clinical signals of denture stomatitis who were excluded from the study group, once they did not present positive results for *Candida *spp. All the 30 selected subjects with health palatal mucosa (15 denture users and 15 with natural teeth) had no growth in culture and any yeast observation at microscopy. They were chosen to attempt a comparison of *Candida *spp. number of colonies between individuals with health palatal mucosa and with denture stomatitis. 

Among the study group patients (*n* = 45) the used time of the same denture varied from three months to 48 years. Systemic diseases were related by 82.6% of them, especially arterial hypertension (53.3%); 77.3% were using some medication, mainly antihypertensive (41.3%). 

The age of these patients was between 27 and 79 years old (mean age 60.76 ± 11.34). There were seven males (15.5%) and 38 females (84.4%). In the presumptive identification of *Candida *spp., only *Candida albicans* was isolated. 

Tables 1, 2, and 3 show the results of the clinical aspects (based in the Newton's classification) and the microbiologic results (total count of CFUs), before and after the treatment with miconazole gel (Group I,Table 1) and with the two forms of propylene glycol Brazilian green propolis: gel (Group II,Table 2) and solution for mouthwash (Group III,Table 3).

The three treatments showed significant statistical results in the reduction or in the complete clinical remission of denture stomatitis and in the decrease or elimination of yeast count. No statistical differences in efficacy were seen among the three groups, either for reduction or remission of palatal erythema (*P* = 0.1069) or to reduction or elimination of CFUs (*P* = 0.9586).

Results of Tables 1, 2, and 3 showed that the number of CFUs was not related with the degree of clinical erythema severity, according to Newton's classification. All the patients who still presented denture stomatitis and some CFUs after treatments were maintained in use of their medications for more 14 days, following the same protocol. All denture wearers examined at baseline for this study (*n* = 85) were invited to an evaluation of their dentures in the Prosthesis Clinic of DOPUC Minas.

## 4. Discussion

One of the limitations of the clinical trials is the impossibility of controlling the medicaments correct use by patients at home. Despite this limitation, the results found in the present study showed that all the products used presented efficacy for denture stomatitis clinical cure or reduction and for number of *Candida albicans* colonies in culture elimination or reduction. Few double-blinded, controlled-randomized clinical-trials studies for topical treatment comparison of *Candida*-associated denture stomatitis between Brazilian green propolis and current medicinal treatment generally done with topical well-known antifungal as miconazole gel [2, 5] have been reported. According to our best knowledge, this is the first study which reports the use of 2.5% propylene glycol extract of Brazilian green propolis for the treatment of *Candida*-associated denture stomatitis, comparing clinical erythema and count of CFUs before and after treatment and that established the best safety Brazilian green propolis concentration through *Candida* inhibition test and that made *Candida *spp. identification. 

Propolis is considered a safe natural bee product and has been extensively used in folk medicine since early times for its pharmaceutical properties and its antiviral, antibacterial, and antifungal properties have been demonstrated in a number of investigations [12, 14, 15]. Only few cases of allergy to propolis were reported, and, up to the present moment, there have been no description of Brazilian green propolis side effects [19, 21]. Systemic antifungals as fluconazole are expensive, and the topical ones, such as nystatin and miconazole, have some side effects as informed in their medical bulls. Due to the increasing resistance to fluconazole and the limited power of action and toxicity of some antifungal drugs, new alternatives in the treatment of denture stomatitis are welcome [26]. 

Good results using ethanolic [11, 23] and propylene glycol vehicles of Brazilian propolis in denture stomatitis treatment [27] have been reported. 

Propylene glycol is a humectant vehicle of common use in pharmacologic formulations, and it is an oral mucosa nonirritant product. In this pilot study propylene glycol extract of Brazilian green propolis was well accepted by the patients, and its nonirritant action makes this formulation reliable. Medications with ethanol vehicle could induce irritation and oral erythema that could modify or disguise the results, if denture stomatitis is still present after the treatment [19]. Patients who received mouthwash propolis form were the most pleased in relation to the taste and the easy way of their treatment in this study. 

For the three groups, there was statistically significant yeast cells count suppression or decrease of the palatal mucosa inflammation, after treatment. However, there was no erythema complete remission or total colonies elimination for all patients, neither for the ones who received miconazole nor for the ones who received propolis. Studies with similar methodology using miconazole gel [2, 25] and ethanolic extract of Brazilian green propolis [23] showed similar results. Nevertheless, Santos et al. [27] reported clinical erythema total remission either by using miconazole gel or Brazilian green propolis gel. There was a female prevalence in this study as may be expected from the epidemiology of this condition [28]. 

An association among CFUs number and palatal erythema severity was expected. In spite of that, there was persistent denture stomatitis in patients who presented decrease or absence of *Candida *spp. colonies after treatment, in the three groups evaluated. On the other hand, some patients had a great number of colonies and less erythema after their treatment. These results can be explained by other predisposing factors as systemic diseases, high consumption of saccharose and denture alterations. This could increase CFUs number, regardless candidiasis presence in those patients. Budtz-Jörgensen and Carlino [5] already described no apparent correlation among extensive mycological events when compared to clinical parameters [9]. 

The lack of CFUs number in culture of 35 out of 85 denture wearers with palatal mucosa erythema, assessed at the start of the study, also reinforced the multifactorial etiology of denture stomatitis. Denture stomatitis presence with or without growth of *Candida *spp. colonies found suggests the need of meticulous investigation in medical history of patients with this lesion to identify associated risk factors. This study results also suggest that microbiological tests for *Candida *spp. detection are needed before starting any kind of antifungal therapy. This conduct avoids useless antifungal therapy. According to Barbeau et al. [6], some well-known risk factors associated with denture stomatitis are prosthesis with old age, maladjustments, instability, broken, worn during sleep, deficient in hygiene, and worn by patients who have some systemic diseases or smoking. However, *Candida albicans*, found here in all the 45 patients of the treatment groups, has been considered as an important factor in the etiology of denture stomatitis [1–3, 5, 7, 10]. 

The presumptive identification of *C. albicans* is mainly obtained through germ-tube test. Lacaz et al. [29] reported that *C. dubliniensis* also makes germ tube, in the same conditions. *C. dubliniensis* is more found in immunological depressed patients, and *C. albicans* is generally associated with denture stomatitis. This data points out the hypothesis that, in this study, the positive germ-tube test showed the presence of *C. albicans*.

*C. albicans* is part of the oral microbiota [30]; however, there was no yeast growth of *Candida *spp. in the swabs of the 30 patients with health palatal mucosa in this study. This fact suggests that either palatal mucosa is not a *Candida* common *habitat* in normal conditions or it is difficult to obtain *Candida* samples in the palate.

## 5. Conclusions

The overall results of this randomized clinical trial showed that propylene glycol Brazilian green propolis has an antifungal activity similar to miconazole, in the *C. albicans* colonies decrease and in the erythema reduction of patients with *Candida*-associated denture stomatitis. Brazilian green propolis that has antifungal properties, is a safe, affordable, and natural product without well-known side effects up to now. Thus, it can be a good alternative in *Candida*-associated denture stomatitis treatment, especially for public health.

## Figures and Tables

**Table 1:** Quantitative evaluation of yeasts (CFUs) and clinical classification of denture stomatitis before and after the treatment with Miconazole gel 2%.

Patient	Age	Gender	*N* CFUs* Before treatment	*N* CFUs** After treatment	Denture stomatitis classification Before treatment*	Denture stomatitis classification After treatment**
1	58	F	20	1	II	Cure
2	79	M	189	79	III	1
3	49	F	12	0	I	Cure
4	66	F	12	0	II	I
5	69	F	19	0	I	Cure
6	73	F	18	0	I	Cure
7	72	F	95	2	I	Cure
8	73	F	15	0	III	I
9	66	F	38	0	II	I
10	65	F	27	1	II	II
11	60	F	226	0	I	I
12	61	F	43	0	I	I
13	27	F	11	1	III	I
14	74	M	62	0	I	Cure
15	45	M	168	0	III	I

*CFUs: colony-forming units.

**Newton's classification (type: 1; 2; 3).

Clinical evaluation before and after treatment—Wilcoxon's test *P* = 0.0007.

Quantitative microbiological evaluation before and after treatment—Wilcoxon's test *P* = 0.0022.

Mean age: 62.47 ± 13.54.

**Table 2:** Quantitative evaluation of yeasts (CFUs) and clinical classification of denture stomatitis before and after the treatment with propylene glycol Brazilian green propolis gel 2.5%.

Patient	Age	Gender	*N* CFUs* Before treatment	*N* CFUs** After treatment	Denture stomatitis classification** Before treatment	Denture stomatitis Classification** After treatment
1	52	F	64	0	I	Cure
2	78	F	78	1	I	Cure
3	61	M	84	0	II	Cure
4	68	F	136	6	I	I
5	30	F	45	4	II	II
6	58	F	288	89	II	I
7	78	F	189	0	II	I
8	72	F	9	0	III	Cure
9	59	F	548	0	III	I
10	66	F	52	24	II	I
11	50	F	58	0	I	Cure
12	46	F	158	33	II	I
13	45	F	472	117	III	I
14	53	F	283	23	III	I
15	50	M	47	0	I	I

*CFUs: colony-forming units.

**Newton's classification (type: 1; 2; 3).

Clinical evaluation before and after treatment—Wilcoxon's test *P* = 0.0007.

Quantitative microbiological evaluation before and after treatment—Wilcoxon's test *P* = 0.0022.

Mean age: 57.73 ± 13.24.

**Table 3:** Quantitative evaluation of yeasts (CFUs) and clinical classification of denture stomatitis before and after the treatment with propylene glycol Brazilian green propolis 24% solution for mouthwash.

Patient	Age	Gender	*N* CFUs* Before treatment	*N* CFUs** After treatment	Denture stomatitis classification Before treatment	Denture stomatitis classification After treatment**
1	66	F	105	0	II	Cure
2	66	M	269	1	III	Cure
3	60	F	14	0	II	I
4	67	M	18	0	I	Cure
5	65	F	12	0	I	Cure
6	68	F	18	0	III	III
7	63	F	226	1	I	Cure
8	65	F	22	1	III	III
9	46	F	19	0	I	I
10	60	F	19	0	III	III
11	60	F	17	0	I	Cure
12	61	F	28	0	I	Cure
13	65	F	12	0	I	Cure
14	63	F	296	0	III	III
15	56	F	113	0	I	Cure

*CFUs: colony-forming units.

**Newton's classification (type: 1; 2; 3).

Clinical evaluation before and after treatment—Wilcoxon's test *P* = 0.0007.

Quantitative microbiological evaluation before and after treatment—Wilcoxon's test *P* = 0.00077.

Mean age: 62.06 ± 5.52.
